# Jacksonian Prize of the Royal College of Surgeons

**Published:** 1861-05

**Authors:** 


JACKSONIAN PRIZE OF THE ROYAL COLLEGE OF SURGEONS.—The subjects of the Jacksonian prize for 1861 are, "The Structure and Diseases of the Lachrymal Passages at the inner side of the Orbit, being those between the Conjunctiva and Nasal Cavity, illustrated by Drawings and Preparations," and "The best Method of effecting the Radical Cure of Inguinal Hernia; explaining the Principle of the Operation adopted, and illustrating the Subject by Cases and Drawings."

